# Participation in Virtual Prehabilitation and Outcomes Following Thoracic Cancer Surgery

**DOI:** 10.1001/jamanetworkopen.2024.4084

**Published:** 2024-03-28

**Authors:** Jun J. Mao, Daniela Molena, Krupali Desai, Raymond E. Baser, Christina Seluzicki, Gaetano Rocco, David Jones

**Affiliations:** 1 Integrative Medicine Service, Department of Medicine, Memorial Sloan Kettering Cancer Center, New York, New York; 2 Department of Epidemiology and Biostatistics, Memorial Sloan Kettering Cancer Center, New York, New York; 3 Thoracic Service, Department of Surgery, Memorial Sloan Kettering Cancer Center, New York, New York

## Abstract

This cohort study evaluates the association of a virtual synchronized prehabilitation program with perioperative outcomes among patients undergoing thoracic cancer surgery.

## Introduction

The American Society of Clinical Oncology (ASCO) clinical guideline recommends prehabilitation exercise for patients with lung cancer undergoing surgery to reduce postoperative complications.^1^ However, the COVID-19 pandemic presented challenges to accessing on-site exercise programs, and limited research has evaluated virtual platforms for prehabilitation programs in cancer settings. This study aimed to evaluate the association of a virtual synchronized prehabilitation program with perioperative outcomes among patients undergoing thoracic cancer surgery.

## Methods

This cohort study followed the STROBE reporting guideline. This study was approved by the institutional review board of Memorial Sloan Kettering Cancer Center under a retrospective protocol. Informed consent was waived because of the retrospective nature of the study.

We conducted a retrospective cohort study at the thoracic oncology surgical department of an academic cancer center from December 2019 to December 2021. Eligible patients were 18 years or older with a thoracic cancer diagnosis and expected thoracic surgery between December 2019 and December 2021. The prehabilitation program offered 2 free weekly 45-minute preoperative virtual mind-body fitness classes with the same content to help these patients prepare for surgery between December 2020 to December 2021.^2^ Patients who attended at least 1 class prior to surgery were included in the analysis (prehabilitation group). We randomly selected 400 control patients who were not approached to enroll in the prehabilitation program from December 2019 to December 2021. Cohorts were stratified by cancer type (lung, esophageal, other) to match the cancer type proportions in the prehabilitation group (±5%). Using electronic medical records, we extracted length of hospital stays (LOS) and whether they had a hospital readmission or urgent care center (UCC) visit within 30 days of discharge.

Descriptive statistics were used to summarize demographic and clinical variables. We compared LOS between prehabilitation, and control groups using a Wilcoxon rank-sum test and compared 30-day hospital readmission and UCC visits using Pearson χ^2^ tests. A multivariable logistic regression model was built to estimate hospital readmission and included covariates associated with the outcome (*P* < .10) in the univariable analyses. All tests were 2-sided, and the significance was set at *P* <.05. Stata version 13.0 (StataCorp) was used for analyses. Data were analyzed from December 2022 to June 2023.

## Results

Among 519 patients analyzed, 119 (median [IQR] age, 70 [65-76] years; 59 [49.6%] female; 67 [56.3%] had lung cancer; 33 [27.7%] had esophageal cancer) were in the prehabilitation group and 400 (median [IQR] age, 69 [62-76] years; 216 [54%] female, 239 [59.8%] had lung cancer; 101 [25.3%] had esophageal cancer) were in the control group (Table 1). Compared with the control group, the prehabilitation group had reduced hospital readmissions (45 of 400 patients [11.3%; 95% CI, 8.5%-14.8%] vs 4 of 119 patients [3.4%; 95% CI, 1.3%-8.7%; *P* = .01). The difference in UCC visits was not statistically significant, with 56 in the control group (14.0%; 95% CI, 10.9%-17.8%) vs 10 in the prehabilitation group (8.4%; 95% CI, 4.6%-15.0%) (*P* = .11). No significant difference in LOS was found between the control and prehabilitation groups. In a multivariable logistic regression, participating in prehabilitation (odds ratio [OR], 0.26; 95% CI, 0.09-0.75; *P* = .01) and having lung as compared with esophageal cancer (OR, 0.46; 95% CI, 0.23-0.92; *P* = .03) were associated with significantly lower odds of readmission (Table 2).

**Table 1.** Patient Characteristics and Outcomes Among Patients Who Participated in the Prehabilitation Program and Those Who Did Not Participate

Characteristics	Participants, No. (%)		*P* value
	Participated in prehabilitation program (n = 119)	Did not participate in prehabilitation program (n = 400)	
Baseline patient characteristics			
Age, median (IQR)	70 (65-76)	69 (62-76)	.80
Sex			
Female	59 (49.6)	216 (54.0)	.40
Male	60 (50.4)	184 (46.0)	
Race			
African American	2 (1.7)	12 (3.0)	.62
Asian	7 (5.9)	31 (7.8)	
White	104 (87.4)	344 (86)	
Other	4 (3.4)	8 (2.0)	
Unknown	2 (1.7)	5 (1.3)	
Ethnicity			
Hispanic or Latino	8 (6.7)	15 (3.8)	.14
Non-Hispanic or Latino	103 (86.6)	370 (92.5)	
Unknown	8 (6.7)	15 (3.8)	
Cancer type			
Lung	67 (56.3)	239 (59.8)	.80
Esophageal	33 (27.7)	101 (25.3)	
Other	19 (12.9)	60 (15.0)	
ASA Score			
2	7 (5.9)	46 (11.5)	.21
3	103 (86.6)	326 (81.5)	
4	9 (7.6)	26 (6.5)	
5	0	2 (0.5)	
BMI, mean (IQR)^a^	28.5 (24.6-31.2)	27.5 (30.4-23.7)	.08
Perioperative outcomes			
Readmissions within 30 d of discharge	4 (3.4)	45 (11.3)	.01
LOS, median (IQR), d^b^	5 (2-8)	4 (2-7)	.12
Urgent care center visit within 30 d of discharge	10 (8.4)	56 (14.0)	.11

Abbreviations: ASA, American Society of Anesthesiologists Physical Status Classification System; BMI, body mass index (calculated as weight in kilograms divided by height in meters squared); LOS, length of hospital stay.

^a^ BMI was available for 377 individuals in the control group and 117 individuals in the prehabiliation group.

^b^ First quartile and third quartile.

**Table 2.** Multivariable Logistic Regression Model of Factors Associated With Readmission Within 30 Days of Discharge

Factor	Readmission within 30 d of discharge, AOR (95% CI)	*P* value
Participation in prehabilitation program		
No	1 [Reference]	NA
Yes	0.26 (0.09-0.75)	.01
Sex		
Male	1 [Reference]	NA
Female	0.71 (0.37-1.34)	.29
Cancer type		
Esophageal	1 [Reference]	NA
Lung	0.46 (0.23-0.92)	.03
Other	0.68 (0.28-1.64)	.39

Abbreviation: AOR, adjusted odds ratio.

## Discussion

Participation in a virtual synchronous prehabilitation program was associated with fewer hospital readmissions within 30 days of discharge compared with not participating. Hospital readmission is an important metric for quality of care and health care costs. Although prior research demonstrated various benefits of prehabilitation on perioperative outcomes,^3^ few studies have explored the association of prehabilitation with hospital readmissions among this population, and those that have, reported either no difference^4,5^ or a difference that was not statistically significant^6^ between groups. Our study contributes additional evidence of virtual prehabilitation programming on perioperative outcomes warranting further investigation.

This study has limitations. Although the baseline characteristics were similar between the 2 groups and we adjusted for relevant confounders, our study was observational, and associations may not be causal. Furthermore, there may be residual confounding because we did not have some clinical variables, such as socioeconomic status. Additionally, this study was conducted in a tertiary cancer center, which limits its generalizability to community settings. Despite these limitations, our virtual prehabilitation program represents a scalable solution to implement ASCO clinical guidelines to improve perioperative outcomes for those undergoing thoracic surgery. Future randomized clinical trials are needed.

## Notes

Supplement.
Data Sharing Statement
