# Supplementary material for: Participation in Virtual Prehabilitation and Outcomes Following Thoracic Cancer Surgery
Source: JAMA Netw Open. 2024 Mar 28;7(3):e244084. doi: 10.1001/jamanetworkopen.2024.4084 (PMC10979307; doi:10.1001/jamanetworkopen.2024.4084)
Supplement: Supplement. — Data Sharing Statement [file jamanetwopen-e244084-s001.pdf]

Data Sharing Statement

Data
Data available: Yes
Data types: Deidentified participant data
How to access data: Data will be available upon request to maoj@mskcc.org
When available: With publication

Supporting Documents
Document types: None

Additional Information
Who can access the data: researchers whose proposed use of the data has been approved
Types of analyses: for a specified purpose
Mechanisms of data availability: with a signed data access agreement
